# Toxicants, Exposome, and Hantavirus Disease: A One Health Perspective

**DOI:** 10.3390/v18060597

**Published:** 2026-05-25

**Authors:** Jose L. Domingo

**Affiliations:** Universitat Rovira i Virgili, Laboratory of Toxicology and Environmental Health, School of Medicine, Sant Llorenç 21, 43201 Reus, Catalonia, Spain; joseluis.domingo@urv.cat

**Keywords:** hantavirus, toxicants, exposome, One Health, emerging viruses, climate change, Andes orthohantavirus

## Abstract

Although hantaviruses have traditionally been considered geographically restricted rodent-borne pathogens, globalization, climate change, ecosystem disruption, and environmental contamination may collectively favor novel transmission scenarios and altered epidemiological patterns. The experience gained during the SARS-CoV-2 pandemic showed the importance of environmental determinants, airborne exposure, and host susceptibility factors in emerging viral diseases. In this context, increasing but still indirect evidence suggests that environmental toxicants and the exposome may modulate susceptibility to hantavirus infection and influence disease severity. The proposed mechanisms include oxidative stress, endothelial dysfunction, pulmonary inflammation, and immune dysregulation, rather than direct causal effects of toxicants on infection itself. This article discusses current knowledge regarding interactions among toxic environmental exposures, climate change, and hantavirus disease, with special emphasis on Andes orthohantavirus (ANDV), the principal hantavirus known to exhibit person-to-person transmission. The article integrates recent evidence within the One Health framework and highlights future research priorities linking environmental toxicology, zoonotic disease ecology, and global environmental change.

## 1. Introduction

The SARS-CoV-2 pandemic demonstrated how emerging viral diseases can rapidly escalate into global health crises, while also revealing the importance of environmental determinants, such as air pollution and climate-related variables, in modulating viral transmission, host susceptibility, and disease severity [1,2]. Although these associations do not imply causation, they provide important insights into how environmental stressors may influence host vulnerability to viral infections.

Hantaviruses have attracted renewed attention due to changing ecological conditions, expanding rodent habitats, environmental degradation, and the increasing potential for atypical transmission events [3,4]. In early May 2026, the WHO reported a cluster of severe respiratory illness among cruise ship passengers, with six laboratory-confirmed hantavirus cases, five suspected cases, and three deaths [5]. This event has revived concerns regarding the epidemic potential of hantaviruses, particularly Andes orthohantavirus (ANDV), the only hantavirus with convincing evidence of person-to-person transmission [6,7].

Hantaviruses are enveloped RNA viruses belonging to the family Hantaviridae. Old World hantaviruses mainly cause hemorrhagic fever with renal syndrome (HFRS), whereas New World hantaviruses produce hantavirus cardiopulmonary syndrome (HCPS), a frequently fatal disease characterized by pulmonary edema, severe respiratory failure, endothelial dysfunction, and capillary leakage [8,9,10].

**Figure 1:**
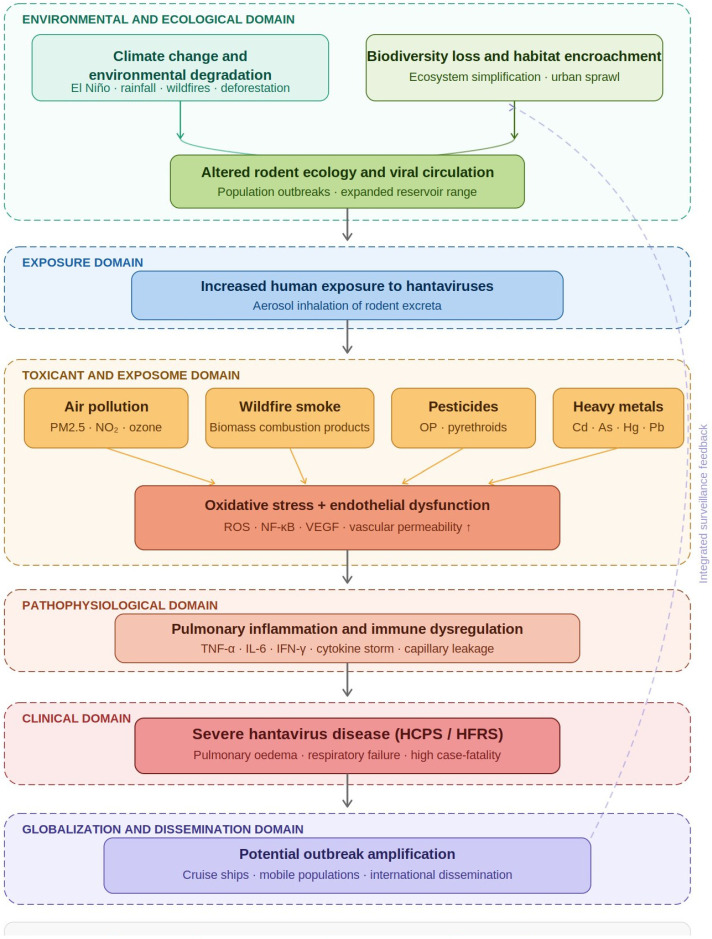
Proposed One Health interactions among toxicants, climate change, and hantavirus disease. ANDV: Andes orthohantavirus; HCPS: hantavirus cardiopulmonary syndrome; PM_2.5_: fine particulate matter; ROS: reactive oxygen species. Key references: climate change and cross-species viral transmission [12]; zoonotic host diversity in human-dominated ecosystems [13]; biodiversity and infectious disease [14]; air pollution and respiratory viral infections [1,21]; toxicants and immune dysregulation [22,23]; hantavirus epidemiology [8,9]; One Health framework [19,20]; exposome [18]; globalization and disease dissemination [5].

Transmission occurs primarily through inhalation of aerosolized excreta from infected rodents. However, increasing attention has been paid to a broader set of environmental, ecological, and anthropogenic modifiers that may influence spillover risk and clinical severity [11]. Climate change, biodiversity loss, urban expansion, deforestation, air pollution, and toxic environmental exposures may alter rodent reservoir dynamics, increase human exposure, and modulate host immune responses [12,13,14,15,16,17]. The concept of the exposome, encompassing the totality of environmental exposures throughout life, offers an important framework for understanding how toxicants and environmental stressors may interact with infectious diseases [18]. Simultaneously, the One Health paradigm recognizes the interconnectedness among human health, animal reservoirs, and environmental conditions in the emergence of zoonotic pathogens [19,20]. The present article examines recent evidence linking toxic environmental exposures, climate change, and hantavirus disease, with particular emphasis on ANDV and its potential implications for future epidemic scenarios. Importantly, this paper adopts a hypothesis-generating approach rather than implying established causal relationships. A schematic representation of these interactions is shown in Figure 1.

## 2. Environmental Toxicants and Hantavirus Disease

Although direct causal evidence remains limited, multiple environmental toxicants induce biological alterations remarkably similar to the pathogenic mechanisms observed during severe hantavirus infection. A defining characteristic of HCPS is endothelial dysfunction with increased vascular permeability. Importantly, pulmonary edema in hantavirus disease appears to result primarily from dysregulated host inflammatory responses rather than direct viral cytotoxicity [24]. High levels of TNF-α, IL-6, IFN-γ, VEGF, and reactive oxygen species (ROS) have been reported in severe disease [25,26]. Comparable inflammatory and oxidative pathways are also activated by several environmental toxicants. The main environmental toxicants, their biological/ecological effects, and their potential implications for hantavirus disease are summarized in Table 1. The relationships should be interpreted as biologically plausible interactions rather than demonstrated causal links.

### 2.1. Air Pollution and Pulmonary Vulnerability

Air pollution has emerged as one of the most important environmental determinants of respiratory viral disease severity. Fine particulate matter (PM_2.5_), ultrafine particles, nitrogen oxides, ozone, and biomass smoke induce oxidative stress, endothelial injury, pulmonary inflammation, and impaired innate immunity [1,21,27,37]. During the COVID-19 pandemic, positive associations between outdoor air pollution and SARS-CoV-2 incidence and severity were found [1,37,38]. By analogy, similar mechanisms may also be relevant for hantavirus infection, although direct epidemiological confirmation is lacking. Particulate pollutants increase alveolar-capillary permeability and activate inflammatory pathways involving NF-κB and cytokine production [28]. These effects closely resemble the pathogenic events responsible for pulmonary edema in HCPS. Therefore, chronic exposure to polluted air can create a vulnerable pulmonary microenvironment favoring severe hantavirus disease. This interpretation remains hypothetical and needs specific investigations. In turn, tobacco smoke can exert analogous effects. Smoking induces oxidative stress, epithelial injury, endothelial dysfunction, and dysregulated antiviral responses [29]. Experimental evidence indicates that cigarette smoke impairs alveolar macrophage activity and interferon-mediated antiviral defenses, mechanisms potentially relevant for hantavirus susceptibility.

### 2.2. Heavy Metals and Immunotoxicity

Heavy metals such as cadmium, arsenic, mercury, and lead are recognized immunotoxicants capable of inducing oxidative stress, mitochondrial dysfunction, and chronic inflammation [22,30,31]. Cadmium deserves particular attention because of its preferential pulmonary accumulation and its association with tobacco smoke exposure. Experimental studies have shown that cadmium increases pulmonary permeability and disrupts endothelial integrity [32]. Such effects could theoretically amplify the vascular leakage characteristic of HCPS.

### 2.3. Pesticides and Occupational Exposures

Agricultural workers in endemic regions are frequently exposed simultaneously to rodent reservoirs and immunotoxic pesticides. Organophosphate and pyrethroid pesticides alter macrophage function, cytokine signaling, and oxidative balance [23]. Although epidemiological evidence remains insufficient to establish causality, pesticide-induced immune dysregulation represents a plausible cofactor influencing disease severity.

## 3. Climate Change and Hantavirus Emergence

Climate change is increasingly recognized as a major driver of emerging infectious diseases [33]. The COVID-19 experience provides a useful precedent for hantaviruses, not as a direct analogy but as an illustration of climate–infection interactions. Modeling studies indicate that climate-driven range shifts in wildlife markedly increase cross-species viral transmission opportunities, including for bat-borne coronaviruses such as SARS-CoV-2 [12]. Altered temperature patterns, changing precipitation regimes, droughts, wildfires, and ecological disruption can modify rodent population dynamics and viral transmission cycles. Rodent reservoir abundance is strongly influenced by environmental conditions affecting food availability and breeding success. Increased precipitation associated with El Niño events has previously been linked to outbreaks of hantavirus disease in the Americas [34]. Climate-driven environmental changes may also expand the geographical distribution of rodent hosts, increasing opportunities for human exposure. Simultaneously, deforestation, habitat fragmentation, urbanization, and biodiversity loss facilitate closer contact between wildlife reservoirs and human populations [14]. Wildfires deserve special attention within this context. In addition to their ecological consequences, wildfire smoke contains high concentrations of PM_2.5_ and toxic combustion products capable of impairing respiratory immunity [36]. Climate-mediated changes in rodent reservoir dynamics, habitat use, and human–rodent contact patterns are likely to modulate hantavirus circulation and the risk of outbreaks [35].

## 4. Andes Orthohantavirus (ANDV): A Particular Concern

ANDV is unique among hantaviruses because it has demonstrated person-to-person transmission, particularly in Argentina and Chile [6]. This characteristic raises concerns regarding epidemic potential under favorable epidemiological conditions. It has been suggested that ANDV exhibits distinct pathogenic and immunological characteristics compared with other hantaviruses [39]. These characteristics include a particularly pronounced cytokine storm with elevated levels of TNF-α, IFN-γ, and IL-6 compared with other New World hantaviruses, as well as more pronounced endothelial dysfunction and vascular leakage [25,39]. Notably, ANDV has been shown to inhibit the type I interferon response through its NSs protein, which antagonizes MAVS signaling, thereby facilitating viral persistence and contributing to immune evasion [7]. These immunological features may collectively explain the higher case fatality rates and greater epidemic potential associated with ANDV relative to other hantavirus species. Although sustained transmission has remained limited, viral persistence in secretions, close-contact transmission, and pulmonary involvement may facilitate secondary transmission events. Climate change could further influence ANDV epidemiology by modifying the distribution of rodent reservoirs such as *Oligoryzomys longicaudatus* [40]. Ecological disturbances in South America, including forest fires, changing precipitation patterns, and habitat alteration, may affect viral circulation and human exposure.

The recent WHO notification involving a cruise ship cluster should be interpreted cautiously, but serves as an example of how globalization and international travel could potentially contribute to rapid dissemination of emerging hantavirus infections [5]. Although sustained human-to-human transmission has not yet been demonstrated outside limited clusters, lessons learned from SARS-CoV-2 emphasize the importance of early vigilance.

## 5. One Health Implications of Hantavirus Emergence

The emergence and re-emergence of hantavirus infections cannot be understood in isolation from broader ecological, climatic, anthropogenic, and social processes. A comprehensive One Health analysis requires examining the multiple intersecting domains through which human, animal, and environmental health interact to shape hantavirus transmission dynamics and disease burden.

### 5.1. Wildlife Reservoirs and Ecosystem Disruption

Rodent ecology is central to understanding hantavirus transmission dynamics. Each hantavirus species is closely associated with a specific rodent host: ANDV with the long-tailed rice rat (*Oligoryzomys longicaudatus*) in southern South America, Sin Nombre virus with the deer mouse (*Peromyscus maniculatus*) in North America, and Hantaan virus with the striped field mouse (*Apodemus agrarius*) in Asia [8,9]. Rodent population dynamics are governed by resource availability, predation, climate variability, and habitat quality, all of which are being increasingly altered by human activities.

Biodiversity loss plays a particularly important mechanistic role. The dilution effect hypothesis proposes that high host diversity reduces zoonotic disease transmission risk by decreasing the probability that a vector or pathogen encounters a competent reservoir host [14]. Conversely, anthropogenic simplification of ecosystems, through deforestation, agricultural intensification, and urban sprawl, tends to favor the proliferation of generalist rodent species that are often the most competent hantavirus reservoirs [13]. Habitat encroachment thus creates not only closer human–rodent contact but also ecological conditions that may amplify viral prevalence within reservoir populations.

### 5.2. Climate Change and Zoonotic Spillover

The El Niño–Southern Oscillation (ENSO) provides a well-documented model for understanding how climate variability drives hantavirus outbreaks. El Niño events increase precipitation across parts of the Americas and Asia, promoting vegetation growth, food abundance, and subsequent rodent population eruptions [34]. The 1993 Four Corners outbreak in the United States and multiple Andean outbreaks have been temporally associated with such climatic cycles. Analyses of hantavirus hemorrhagic fever in Central China showed that interannual cycles of outbreaks are significantly controlled by temperature and rainfall patterns [15]. Climate change is projected to intensify ENSO variability and alter global rainfall patterns in ways that can increase the frequency and geographic scope of rodent outbreaks associated with zoonotic spillover [12,33]. Large wildfires destroy rodent habitats and displace reservoir populations into human settlements, simultaneously generating biomass smoke that impairs pulmonary immune defenses [36]. The convergence of ecological displacement and respiratory vulnerability represents a particularly concerning compound risk within the One Health framework.

### 5.3. Anthropogenic Pollution and Infectious Vulnerability

Air pollution occupies a central position in the relationship between anthropogenic contamination and infectious disease vulnerability. Chronic exposure to PM_2.5_, nitrogen dioxide, ozone, and other pollutants induces persistent low-grade pulmonary inflammation, impairs mucociliary clearance, and dysregulates innate immune responses including interferon signaling pathways [21,27]. These pathophysiological alterations closely parallel the host factors that determine severity of HCPS, particularly endothelial dysfunction and uncontrolled inflammatory cytokine responses [24]. The COVID-19 pandemic provided substantial epidemiological evidence that populations residing in highly polluted regions suffered disproportionate disease severity, suggesting a generalizable mechanism of pollution-mediated infectious vulnerability [1,38].

Industrial contamination compounds these risks through heavy metals and persistent organic pollutants that accumulate in human tissues and induce chronic immunotoxicity. In industrialized and mining regions of South America, where ANDV is endemic, communities may experience concurrent exposure to rodent reservoirs and environmental contaminants, creating a particularly adverse combination of exposure risks [41]. The exposome concept, encompassing the totality of chemical, biological, physical, and psychosocial exposures across the life course, provides a suitable framework for integrating these multiple dimensions of environmental vulnerability and understanding their cumulative impact on host susceptibility to emerging viral pathogens [18].

### 5.4. Globalization and Travel-Associated Transmission

The May 2026 WHO notification of a hantavirus cluster among cruise ship passengers illustrates how modern travel ecosystems can serve as amplifiers of geographically restricted zoonotic pathogens [5]. Cruise ships concentrate mobile populations from diverse geographic and immunological backgrounds in confined spaces, facilitate complex supply chains involving multiple ports of call, and generate conditions in which both initial exposure and secondary person-to-person transmission could theoretically occur. For ANDV specifically, the documented capacity for person-to-person transmission under close-contact conditions makes such congregate settings potentially conducive to cluster formation, even though transmission chains have historically remained limited [6].

Beyond maritime tourism, international travel broadly contributes to the potential for geographic dissemination of hantavirus infections. Endemic regions in Argentina, Chile, Brazil, Paraguay, and Bolivia receive substantial volumes of adventure and ecotourism activities that may bring visitors into habitats where rodent exposure risk is heightened. Returning travelers with incubating hantavirus infections may present to healthcare systems in non-endemic countries where clinical recognition is limited, risking diagnostic delays and potentially exposing healthcare workers in settings where awareness of person-to-person transmission risk is insufficient.

### 5.5. Integrated Surveillance Systems

Addressing the complex, multi-domain risks described above requires surveillance systems that transcend traditional human disease reporting. Environmental monitoring of rodent populations, including real-time tracking of abundance, spatial distribution, infection prevalence, and population genetics, constitutes a basic element of early warning systems for hantavirus emergence. Such programs, currently operating in limited form in Chile, Argentina, and the United States, have demonstrated that rodent seroprevalence data can precede and predict human case clusters [9].

Wastewater-based epidemiology (WBE) emerged as a powerful public health tool during the SARS-CoV-2 pandemic [42]. It warrants consideration for application to hantavirus surveillance, particularly in settings with congregate populations such as cruise ships, military installations, and refugee camps [43]. Although the predominantly respiratory route of ANDV transmission differs from the fecal–oral dynamics most amenable to WBE, urinary excretion of hantavirus antigens and RNA has been documented in some infected individuals, suggesting potential signal detection through environmental sampling. Hantavirus RNA has been detected in urine of experimentally infected animals as well as in patients with HFRS, suggesting that renal shedding may provide a detectable environmental signal in endemic or outbreak settings [44,45]. The potential application of WBE to hantavirus surveillance therefore merits further experimental validation, particularly in areas with documented rodent reservoir activity. Integration of wildlife surveillance, environmental sampling, climate data, and human case reporting into unified digital platforms represents an achievable goal that would substantially improve public health preparedness for hantavirus emergence scenarios.

## 6. One Health and Exposome Perspectives

The interaction among environmental toxicants, climate change, ecosystem disruption, and zoonotic infections strongly supports application of the One Health framework. The exposome concept provides an additional integrative perspective by considering cumulative environmental exposures throughout life [18]. Individuals exposed chronically to polluted air, tobacco smoke, occupational toxicants, psychosocial stressors, and poor environmental conditions may exhibit increased vulnerability to severe infectious diseases.

Future research on hantavirus disease should therefore integrate environmental biomonitoring, geospatial exposure assessment, rodent ecology, climate modeling, toxicological biomarkers, immune profiling, and viral genomics. Such multidisciplinary approaches can improve identification of vulnerable populations and prediction of outbreak risk.

## 7. Limitations

Several limitations warrant acknowledgment. First, no direct epidemiological studies have examined associations between environmental toxicants and hantavirus disease severity. Therefore, mechanistic plausibility is drawn from other viral infections. Second, animal model data specifically investigating toxicant pre-exposure followed by hantavirus challenge are lacking. Third, ecological fallacy risks preclude individual-level causal inference from population-level associations. Finally, the exposome framework is methodologically challenging to operationalize in zoonotic disease ecology. Accordingly, this paper is aimed at generating hypotheses and guiding future research rather than establishing causal relationships.

## 8. Conclusions

The recent hantavirus cluster associated with cruise ship travel has renewed concerns regarding the epidemic potential of emerging hantavirus infections, particularly ANDV. Although hantaviruses remain primarily rodent-borne zoonoses, growing evidence suggests that environmental toxicants, climate change, and ecological disruption may modulate, rather than determine, both transmission dynamics and disease severity.

Air pollution, tobacco smoke, pesticides, and heavy metals induce biological effects overlapping substantially with the pathogenic mechanisms of severe hantavirus disease, including oxidative stress, endothelial dysfunction, pulmonary inflammation, and immune dysregulation. However, direct causal links remain insufficiently demonstrated. Climate change may additionally alter rodent ecology, expand reservoir habitats, increase wildfire-related pollution, and facilitate novel exposure scenarios.

Integrating toxicology, exposome research, climate science, and infectious disease biology within the One Health framework should be essential for understanding future hantavirus emergence and preparing for potential epidemic threats. The central message of this article is not that toxicants directly cause hantavirus infection, for which direct evidence remains limited, but rather that environmental degradation, climate change, toxic exposures, rodent ecology, and globalization can collectively modulate the emergence, transmission dynamics, and severity of hantavirus disease within a One Health framework. From a practical standpoint, the findings discussed in this article support the following recommendations: (1) reduction in environmental pollutant exposures in hantavirus-endemic communities, particularly those involving PM_2.5_, heavy metals, and pesticides, through evidence-based regulatory and public health measures; (2) integration of rodent population surveillance with environmental contamination monitoring in high-risk endemic regions; (3) strengthening of wastewater-based epidemiological systems in congregate settings as early-warning tools; (4) targeted health education for agricultural and ecotourism workers in endemic areas; and (5) development of multi-country prospective studies that co-assess toxicant exposures and hantavirus disease severity to generate the direct epidemiological evidence that this field currently lacks.

## Figures and Tables

**Table 1:** Potential interactions among toxicants, climate change, and hantavirus disease.

Factor	Principal Biological/Ecological Effects	Potential Implications for Hantavirus Disease	Key References
PM_2.5_ and air pollution	Oxidative stress, endothelial dysfunction, pulmonary inflammation	Increased susceptibility to severe HCPS	[1,21,27,28]
Tobacco smoke	Impaired innate immunity, epithelial injury	Enhanced pulmonary vulnerability	[29]
Heavy metals (Cd, As, Hg, Pb)	Immunotoxicity, mitochondrial dysfunction	Potential amplification of vascular leakage	[22,30,31,32]
Pesticides	Cytokine dysregulation, oxidative stress	Altered antiviral responses	[23]
Climate change	Rodent habitat expansion, altered precipitation	Increased viral circulation	[12,33,34,35]
Wildfires	Biomass smoke exposure and ecosystem disruption	Combined toxicological and ecological effects	[36]
Deforestation/urbanization	Increased human–rodent contact	Enhanced zoonotic transmission	[13,14]
Globalization/International travel	Rapid geographic dissemination; congregation of diverse populations in confined spaces (e.g., cruise ships)	Potential for atypical clusters and secondary transmission events, particularly for ANDV	[5,6]

## Data Availability

No new data were created or analyzed in this study. Data sharing is not applicable to this article.
